# Exploring the Gut‐Prostate Axis: Microbial Signatures Linked to Prostate Volume and Bladder Function

**DOI:** 10.1002/pros.70160

**Published:** 2026-03-18

**Authors:** Jonathan Surber, Marie Lork, Yasser Morsy, Michael Scharl, Daniel Stephan Engeler, Janine Langenauer, Lukas John Hefermehl, Anna Ebner, Basil Kaufmann, Manuela Hunziker, Daniel Eberli, Uwe Bieri, Cédric Poyet

**Affiliations:** ^1^ Department of Urology University Hospital of Zurich University of Zurich Zurich Switzerland; ^2^ Department of Gastroenterology and Hepatology University Hospital of Zurich University of Zurich Zurich Switzerland; ^3^ Department of Urology Cantonal Hospital of St. Gallen, School of Medicine University of St. Gallen St. Gallen Switzerland; ^4^ Department of Surgery Division of Urology, Kantonsspital Baden Baden Switzerland; ^5^ Department of Urology Stadtspital Triemli Zurich Switzerland

**Keywords:** benign prostate hyperplasia, BPH, microbiome, prostate volume, residual bladder volume

## Abstract

**Background:**

Benign prostatic hyperplasia (BPH) is a common urologic condition in aging men, often linked to systemic inflammation and metabolic dysfunction. Emerging evidence suggests that the gut microbiome may contribute to prostate health and disease. Here we aim to explore potential associations between gut microbiota composition and clinical parameters, such as prostate volume (PV) and residual bladder volume (RBV).

**Methods:**

This cross‐sectional study analyzed stool samples from 28 patients undergoing transurethral surgery. Gut microbiota composition was analyzed using 16S rRNA gene sequencing. Patients were stratified into groups based on PV ( ≤ 40 mL vs. > 40 mL) and RBV ( ≤ 100 mL vs. > 100 mL). α‐diversity (Chao1 and Shannon indices) and β‐diversity (Jaccard distance) were calculated. Linear discriminant analysis effect size (LEfSe) was used to identify differentially abundant taxa between groups.

**Results:**

No significant differences in gut microbial α‐ or β‐diversity were observed between groups stratified by PV or RBV. Nevertheless, several specific bacterial taxa showed significant variation between groups. *Methanobrevibacter smithii* was markedly less abundant in patients with PV > 40 mL (*p* < 0.01). Similarly, patients with high RBV ( ≥ 100 mL) exhibited distinct gut microbial profiles compared to those with lower RBV, characterized by a reduced abundance of *Collinsella* and an increased abundance of *Gastranaerophilales* (both *p* < 0.01).

**Conclusion:**

Our findings suggest that while overall gut microbial diversity may remain stable, specific taxa are associated with prostate and bladder phenotypes, supporting the concept of a gut‐prostate axis. Future research should focus on longitudinal studies to investigate how gut (and urinary) microbiota evolve alongside BPH and/or LUTS over time, with the goal of determining whether microbial signatures could serve as early indicators for symptomatic BPH.

## 1 Introduction

Benign prostatic hyperplasia (BPH) is defined by the American Urological Association (AUA) as a histologic diagnosis referring to the proliferation of smooth muscle and epithelial cells within the prostatic transition zone [1]. It often also presents with symptoms, such as a weakened urine stream, increased frequency of urination, delayed start of urination, post‐void dribble, and nocturia. These symptoms are often grouped together as lower urinary tract symptoms (LUTS). Even though LUTS can also be triggered by other conditions, such as urinary tract infections or diabetes, BPH is the most common cause [2]. The incidence of BPH increases with age, affecting over 50% of men by age 60, making it the most common urologic disease among elderly men [3]. In 2021, the age‐standardized incidence rate (ASIR) of BPH was 326.12 per 100,000 people, the age‐standardized prevalence rate (ASPR) was 2782.92 per 100,000, and the age standardized disability adjusted life years (DALY) rate was 55.12 per 100,000. Looking ahead, the global number of BPH cases is expected to grow. From 2022 to 2035, the incidence is projected to rise from 962.4 to 998.6 per 100,000, and the prevalence from 7878.7 to 8620.6 per 100,000 people [4].

Currently, several mechanisms have been proposed regarding the cellular and molecular mechanisms underlying the pathology of BPH. Genetic factors, such as increased predicted expression of the ETV4 gene, have been linked to a higher risk of BPH [5], while somatic alterations (e.g., Y chromosome loss) and hormonal influences—particularly dihydrotestosterone (DHT)‐driven prostate growth and estrogen–testosterone imbalance with aging—also play key roles [5, 6, 7]. Chronic inflammation represents another major contributor, as patients with BPH and LUTS exhibit marked inflammatory cell infiltration in prostatic tissues, accompanied by collagen remodeling characterized by more aligned fibers [8, 9]. Although the trigger for this inflammation remains unclear, growing evidence links the human microbiome to inflammatory processes [10]. The gut microbiome, in particular, has been associated with diverse diseases ranging from fatty liver and inflammatory bowel disease to cardiovascular and chronic kidney diseases [11, 12, 13, 14, 15, 16]. Although anatomically distinct, increasing evidence suggests that the gut and urogenital systems are functionally connected, with studies reporting associations between gut microbial composition and prostate growth, BPH, and even prostate cancer [17, 18, 19, 20]. Given the microbiome's role in regulating systemic inflammation—illustrated by associations between low fiber intake, altered gut microbial composition, and elevated C‐reactive protein (CRP) levels [21]—it is plausible that alterations in gut microbiota could contribute to the chronic inflammation observed in BPH.

Collectively, increasing evidence suggests that the gut microbiome is linked to various aspects of prostate health, including prostate size, inflammation, and overall metabolic status. However, despite these emerging insights, the potential influence of the gut microbiome on BPH remains largely unexplored. A deeper understanding of these microbial–prostatic interactions could open new therapeutic avenues, such as fecal microbiota transplantation (FMT) to restore microbial balance, probiotic interventions, or targeted antibiotic strategies aimed at modulating bacteria associated with inflammation and prostate growth. In this cross‐sectional study, we therefore investigated the stool microbiome composition of patients undergoing transurethral surgery, focusing on its associations with prostate volume (PV) and residual bladder volume (RBV) as key clinical indicators of BPH and LUTS severity.

## 2 Methods

### 2.1 Patient Cohort and Sample Acquisition

For this study, we used samples collected and biobanked as part of the SILENT‐EMPIRE study, the study protocol of which has been previously reported [22]. For the current cohort (*n* = 28), we included patients diagnosed with LUTS without urological or gastrointestinal malignancy undergoing non‐oncological transurethral bladder surgery or transurethral resection of the prostate. Individuals were excluded if they had received antibiotic treatment in the past month, undergone immuno‐/chemotherapy within the last 6 months, or were on immunosuppressive therapy. Other exclusion criteria include major medical, neoplastic (except skin cancer), surgical, or psychiatric conditions requiring ongoing management, significant gastrointestinal or bladder disorders, past major intestinal surgery, or bladder augmentation surgery. The stool samples were collected as part of the SILENT‐EMPIRE study using the fecal sample collection kit (OMNIgene•GUT | OM‐200, DNA genotek). Patients received instructions on proper sample collection at home and for shipment. Fecal samples were stored at −80°C in a biobank until further analysis.

### 2.2 Patient Data Acquisition

Patient data were gathered from the clinical information system of the University Hospital of Zurich, Cantonal Hospital of St. Gallen and Baden and stored in the REDCap database (Research Electronic Data Capture, Vanderbilt University, Nashville, TN, USA, v 9.3.8), an electronic data capture software.

### 2.3 Metagenome Analysis

The metagenome in fecal samples was analyzed by 16S rRNA gene sequencing by Microsynth AG (Balgach, Switzerland) as described previously [23]. Briefly, total DNA was isolated using the ZymoBiomics DNA Mini Kit (ZymoResearch) according to the manufacturer's instructions. DNA concentration was measured using Quant‐iT PicoGreen dsDNA Assay Kit (ThermoFisher), and integrity was checked by agarose gel electrophoresis. Libraries were generated with the Nextera XT DNA Library Preparation Kit (Illumina) using two‐step PCR amplification with the primer set 515 F × 806 R (V3‐V4 region of 16S rRNA) [24]. After PCR product purification, quantification, and equimolar pooling, paired‐end sequencing (2 × 250 bp) was performed on an Illumina MiSeq platform.

### 2.4 Bioinformatics Analysis

QIIME2 was used to analyze the data. After checking data quality, DADA2 was used for denoising to merge the paired reads and generate amplicon sequence variants (ASVs) to ensure sufficient depth for capturing most features [25]. An optimal sampling depth was selected by performing alpha rarefaction analysis to evaluate α‐ or β‐diversity, resulting in a setting cut‐off of 19,000 reads per sample. Taxonomy was assigned to ASVs using the classify‐learn Naïve Bayes classifier against the pre‐trained Naïve Bayes silva‐138‐99‐nb‐classifier trained against Silva (release 138) full‐length sequences [26].

### 2.5 Statistics

**Figure 1:**
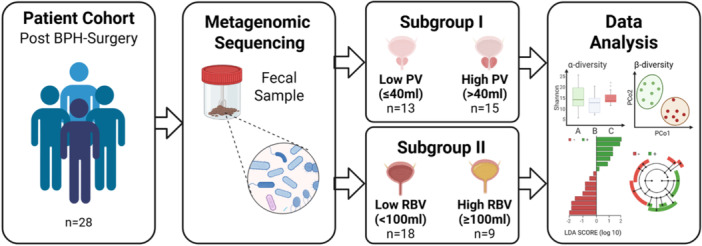
Overview of study design and analysis workflow. Twenty‐eight post‐transurethral surgery patients were enrolled, and fecal samples were analyzed using 16S rRNA gene sequencing. Patients were stratified by prostate volume (PV ≤ 40 mL vs. > 40 mL; Subgroup I) and residual bladder volume (RBV < 100 mL vs. ≥ 100 mL; Subgroup II). Microbial diversity (α‐ and β‐diversity) and taxonomic composition were assessed using standard bioinformatics approaches, including LEfSe. Figure created with BioRender. [Color figure can be viewed at wileyonlinelibrary.com]

For microbiome analysis (16S rRNA sequencing), α‐diversity (species diversity within samples) and β‐diversity (species diversity between samples) were assessed across groups. α‐diversity was evaluated using the Shannon, inverse Simpson, Gini–Simpson, and Chao1 index indices and tested for significance using the Wilcoxon test. β‐diversity was assessed using Bray–Curtis dissimilarity, Jaccard distance, unweighted UniFrac, and weighted UniFrac. Differences between groups were visualized using principal component analysis (PCA). Microbiome composition differences were analyzed using PERMANOVA and ANOSIM. Microbial taxa differing between subgroups were first identified using Linear Discriminant Analysis Effect Size (LEfSe) an exploratory approach for detecting differentially abundant features across groups. Given the limited cohort size, the results should be interpreted as hypothesis‐generating rather than confirmatory. To assess specific pairwise differences in taxa abundance between subgroups, the Wilcoxon rank‐sum test was subsequently applied. Patients were stratified into subgroups based on different clinical parameters. First, they were divided according to PV into a low PV group ( ≤ 40 mL, *n* = 13) and a high PV group ( > 40 mL, *n* = 15). A cutoff of 40 mL was chosen to stratify PV based on its relevance to both clinical decision‐making and previous literature identifying this threshold as indicative of moderate to significant prostatic enlargement [27, 28]. While 30 mL may serve as a general marker for early enlargement, a volume exceeding 40 mL has been associated with more pronounced LUTS, greater bladder outlet obstruction, and the need for medical or surgical intervention [27, 28, 29, 30, 31]. This threshold aligns with clinical scenarios where therapeutic strategies, such as α‐blocker and 5‐α‐reductase inhibitor therapy or surgical consideration, are typically evaluated. The choice of threshold often depends on the specific clinical context and the parameters being assessed [32]. RBV was also used for stratification, as it represents a clinically relevant functional parameter routinely assessed in the evaluation of patients with lower urinary tract dysfunction. Elevated RBV reflects impaired bladder emptying, which may be influenced by bladder outlet obstruction, as well as other factors; a value of ≥ 100 mL is commonly applied as a cutoff [33, 34]. Accordingly, patients were classified into a low RBV group (< 100 mL, *n* = 18) and a high RBV group (≥ 100 mL, *n* = 9). Further subgrouping was performed based on body mass index (BMI ≤ 25 kg/m², *n* = 11 vs. BMI > 25 kg/m², *n* = 17) and age (≥ 65 years, *n* = 15 vs. < 65 years, *n* = 13). Statistical analysis and result visualization was performed using R (R‐Foundation, Vienna, Austria, v 4.1.0) and GraphPad (GraphPad Software Inc., v 10.2.3). A graphical overview figure of the research setup (Figure 1) was created in Biorender.com.

## 3 Results

### 3.1 Cohort Characteristics

We conducted metagenomic analyses of fecal samples to explore associations between the gut microbiome and prostatic hyperplasia (Figure 1). The study cohort consisted of 28 male patients undergoing transurethral surgery, for whom baseline characteristics—including age, body mass index (BMI), alcohol consumption, smoking status, and RBV—were recorded (Table 1). Patients were stratified by PV into a low PV group (≤ 40 mL) and a high PV group (> 40 mL). No statistically significant differences were observed between groups with respect to age, BMI, alcohol consumption, smoking status, or RBV (Table 1). Among the baseline characteristics, only prostate‐specific antigen (PSA) levels differed significantly, with higher values observed in patients with larger PVs, consistent with established associations reported in the literature (Table 1) [35].

**Table 1:** Baseline characteristics of patients.

Patient characteristics	*N*	Overall *N* = 28^a^	Low PV *N* = 13^a^	High PV *N* = 15^a^	*p*‐value^b^
Age (years)	28	66.0 [59.0; 73.0]	62.0 [58.0; 73.0]	66.0 [60.0; 72.0]	0.853
Alcohol consumption	28	9 (32%)	6 (46%)	3 (20%)	0.228
Smoking Status	28				0.655
Never		21 (75%)	11 (85%)	10 (67%)	
Former		6 (21%)	2 (15%)	4 (27%)	
Smoker		1 (3.6%)	0 (0%)	1 (6.7%)	
BMI (kg/m^2^)	28	26.0 [24.5; 28.5]	26.0 [26.0; 27.0]	25.0 [24.0; 29.0]	0.501
Adiposity	28	4 (14%)	3 (23%)	1 (6.7%)	0.311
PSA (ng/mL)	28	2.00 [1.10; 3.71]	1.21 [0.97; 2.00]	3.30 [1.90; 5.83]	**0.010**
RBV (mL)	27	40 [12; 125]	30 [12; 125]	43 [30; 110]	> 0.9
PV (mL)	28	44 [38; 75]	35 [32; 40]	70 [55; 91]	**< 0.0001**

*Note:* Overview of clinical characteristics of the patient cohort stratified by prostate volume ( ≤ 40 mL vs. > 40 mL). Continuous variables are summarized as median [Q1; Q3] and categorical variables as *n* (%). Group comparisons were conducted using the Wilcoxon rank‐sum test for continuous variables and Fisher's exact test for categorical variables. Bold values indicate statistically significant.

Abbreviations: BMI, body mass index; PSA, prostate‐specific antigen; PV, prostate volume; RBV, residual bladder volume.

^a^Median [Q1; Q3]; *n* (%).

^b^Wilcoxon rank‐sum test; Fisher's exact test.

### 3.2 Gut Microbial Composition and Diversity in Relation to Prostate Volume

**Figure 2:**
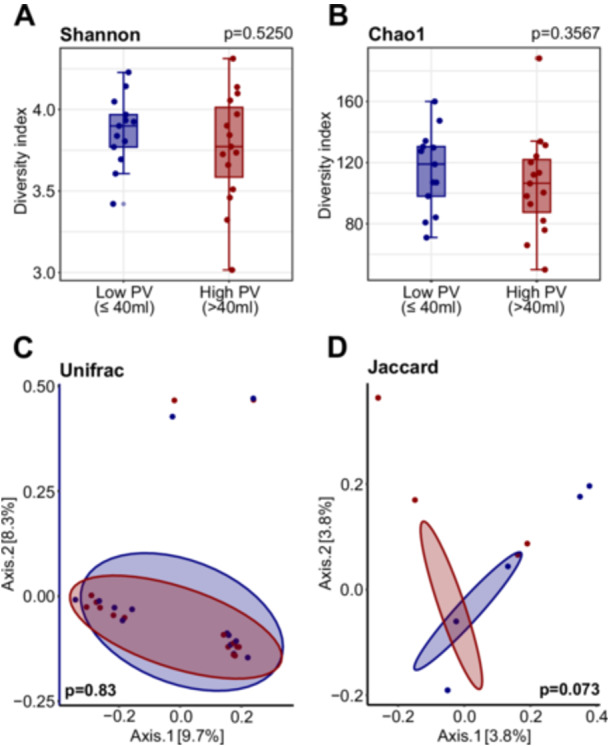
Gut microbial diversity in patients with low and high prostate volume (PV). (A,B) Within‐sample diversity: α‐diversity of gut microbiota in low (PV ≤ 40 mL, *n* = 13) and high (PV > 40 mL, *n* = 15) PV groups, estimated using Chao1 (A) and Shannon (B) indices. All individual data points are shown, with outliers indicated. Statistical differences between groups were assessed using the Wilcoxon rank‐sum test. (C,D) Between‐sample diversity: β‐diversity was assessed using unweighted UniFrac (C) and Jaccard (D) distances and visualized via principal component analysis (PCA). Statistical significance was evaluated using permutational multivariate analysis of variance (PERMANOVA). [Color figure can be viewed at wileyonlinelibrary.com]

To assess differences in gut microbial composition associated with PV, stool samples from patients with low and high PV were analyzed using 16S rRNA gene sequencing. Microbial α‐diversity was evaluated with the Shannon, Chao1, Gini–Simpson, and Inverse Simpson indices, while β‐diversity was assessed with Jaccard, Bray–Curtis, and weighted and unweighted UniFrac distances (Figure 2, Supporting Information Table S1). No statistically significant differences in α‐diversity were observed between patients with PV ≤ 40 mL and > 40 mL (Chao1 *p* = 0.3567; Shannon *p* = 0.5250; Gini–Simpson *p* = 0.5250; Inverse Simpson *p* = 0.5250) (Figure 2A,B, Supporting Information Table S1). Likewise, β‐diversity analysis based on Jaccard distances and visualized by PCA showed no distinct clustering between groups (Jaccard *p* = 0.073, Bray–Curtis *p* = 0.104, weighted UniFrac *p* = 0.721, and unweighted UniFrac *p* = 0.830) (Figure 2C,D, Supporting Information Table S1).

We next assessed the overall gut microbial composition across taxonomic levels (phylum, genus, and species). Substantial heterogeneity in bacterial abundances was observed between individual patient samples (Supporting Information: Figure S1A,B, Table S2). Interestingly, older patients (≥ 65 years) exhibited significantly lower abundances of *Ruminococcus* and *Butyricicoccus*, bacterial genera previously described to decrease with age (*p* = 0.0191 and 0.0228, respectively) (Supporting Information Figure S1C,D) [32]. Detecting these well‐established associations, even in a small cohort, supports the validity of our analytical strategy and underscores its potential to reveal novel microbiome differences across the other clinical subgroupings.

**Figure 3:**
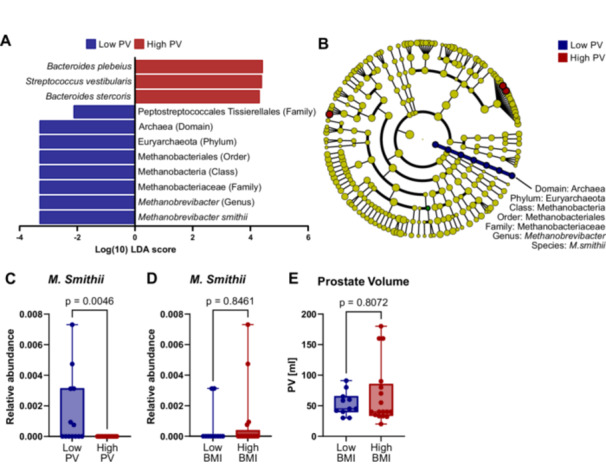
Differentially abundant gut microbiota in patients with low and high prostate volume (PV). (A,B) LEfSe analysis identified taxa differing between the low (PV ≤ 40 mL, *n* = 13) and high (PV > 40 mL, *n* = 15) PV groups. Red indicates taxa enriched in the low PV group, and green indicates taxa enriched in the high PV group. (A) Histogram of logarithmic linear discriminant analysis (LDA) scores for taxa with significant intergroup differences (log10[LDA] > 2, *p* < 0.05). (B) Cladogram showing the taxonomic hierarchy and relative abundance of significantly enriched taxa. Circle size reflects relative abundance. (C,D) Relative abundance of *Methanobrevibacter smithii* in patients stratified by PV ( ≤ 40 mL vs. > 40 mL, C) and body mass index (BMI ≤ 25 kg/m², *n* = 11 vs. BMI > 25 kg/m², *n* = 17, D). Box plots display minimum to maximum values with median. All data points are shown. Statistical significance was assessed using the Mann–Whitney test. [Color figure can be viewed at wileyonlinelibrary.com]

To identify microbial taxa differentially abundant between PV subgroups, LEfSe was performed. The analysis revealed distinct microbial features across multiple taxonomic levels that significantly differed between the two groups. At the species level, *Bacteroides stercoris, Streptococcus vestibularis*, and *Bacteroides plebeius* were significantly more abundant in the high PV group, whereas *Methanobrevibacter smithii* was decreased in patients with high PVs (Figure 3A). Interestingly, cladogram visualization of the LEfSe results showed that *M. smithii* was not only differentially abundant at the species level but that taxa throughout its entire taxonomic lineage were downregulated in patients with high PVs, spanning all hierarchical levels from domain to species (Domain: Archaea; Phylum: Euryarchaeota; Class: Methanobacteria; Order: Methanobacteriales; Family: Methanobacteriaceae; Genus: *Methanobrevibacter*; Species: *Methanobrevibacter smithii*) (Figure 3B). Univariate analysis confirmed that *M. smithii* was significantly less abundant in patients with larger PVs compared to those with smaller prostates (*p* = 0.0046) (Figure 3C).

Given that *M. smithii* has been associated with weight gain in animal models [36] and that obesity in men is linked to a higher risk of developing BPH [37], we sought to explore whether *M. smithii* abundance correlated with body weight in our cohort. To this end, patients were stratified by BMI into normal weight (< 25 kg/m², *n* = 11) and overweight (≥ 25 kg/m², *n* = 17) groups. We then compared both *M. smithii* abundance and PV between these BMI subgroups. No statistically significant differences were observed for neither *M. smithii* abundance (*p* = 0.8461) nor PV (*p* = 0.8027) across BMI groups, suggesting that, within this cohort, body weight did not appear to be linked to either microbial abundance or prostate size (Figure 3D,E).

### 3.3 Gut Microbiome Composition in Relation to Residual Bladder Volume

While PV provides a direct anatomical measure of BPH, RBV reflects a functional urological parameter associated with impaired bladder emptying. To explore whether the gut microbiome is associated with symptom burden in addition to prostate size, patients were stratified into low RBV (< 100 mL; *n* = 18) and high RBV (≥ 100 mL; *n* = 9) groups, and differences in microbial diversity were analyzed.

**Figure 4:**
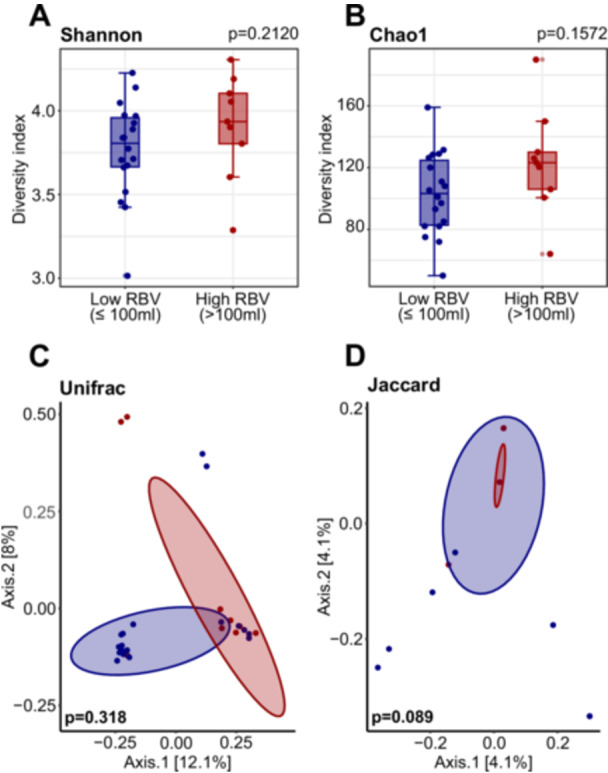
Gut microbial diversity in patients with low and high residual bladder volume (RBV). (A,B) Within‐sample diversity: α‐diversity of gut microbiota in the low (RBV < 100 mL, *n* = 18) and high (RBV ≥ 100 mL, *n* = 9) RBV groups, estimated using Chao1 (A) and Shannon (B) indices. All individual data points are shown, with outliers indicated. Statistical differences between groups were assessed using the Wilcoxon rank‐sum test. (C,D) Between‐sample diversity: β‐diversity was assessed using unweighted UniFrac (C) and Jaccard (D) distances and visualized via principal component analysis (PCA). Statistical significance was evaluated using permutational multivariate analysis of variance (PERMANOVA). [Color figure can be viewed at wileyonlinelibrary.com]

No significant differences were observed between the two groups in intestinal microbiome diversity, either in α‐diversity (Chao1 *p* = 0.1572; Shannon *p* = 0.2120; Gini–Simpson *p* = 0.2750; Inverse Simpson *p* = 0.27450; Figure 4A,B, Supporting Information Table S1) or β‐diversity (Jaccard *p* = 0.089, Bray–Curtis *p* = 0.09, weighted UniFrac *p* = 0.996, and unweighted UniFrac *p* = 0.318; Figure 4C,D, Supporting Information Table S1), suggesting that RBV, as a functional clinical measure of bladder emptying, is not associated with major shifts in overall gut microbial diversity in this cohort.

**Figure 5:**
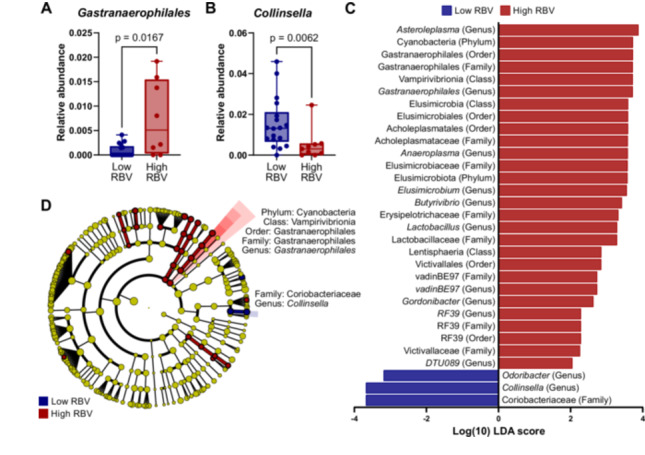
Differential enrichment of gut bacterial taxa in patients with low and high residual bladder volume (RBV). (A,B) Relative abundance of the genera *Collinsella* (A) and *Gastranaerophilales* (B) between the low and high RBV groups. Box plots display median, interquartile range, and individual values. All data points are shown. Statistical significance was assessed using the Mann–Whitney test. (C,D) LEfSe analysis identified taxa differing between the low (RBV < 100 mL, *n* = 18) and high (RBV ≥ 100 mL, *n* = 9) RBV groups. Red indicates taxa enriched in the low RBV group, and green indicates taxa enriched in the high RBV group. (C) Histogram of logarithmic linear discriminant analysis (LDA) scores for taxa with significant intergroup differences (LDA > 2, *p* < 0.05). (D) Cladogram showing the taxonomic hierarchy and relative abundance of significantly enriched taxa. Circle size reflects relative abundance. [Color figure can be viewed at wileyonlinelibrary.com]

Looking at specific taxonomic differences between subgroups, univariate analysis at the species level did not identify any taxa with significant differences between low and high RBV groups. At the genus level, however, several taxa showed significant associations with RBV. Specifically, *Gastranaerophilales* was enriched in patients with high RBV (*p* = 0.0167; Figure 5A), whereas *Collinsella* was more abundant in the low RBV group (*p* = 0.0062; Figure 5B). LEfSe analysis confirmed these findings, highlighting the same genera as enriched in the corresponding RBV subgroups (Figure 5C). Cladogram visualization further illustrated the hierarchical relationships of these taxa, showing that the corresponding higher taxonomic ranks were regulated in the same direction as their respective genera (Figure 5D). Specifically, *Gastranaerophilales* was enriched together with its broader lineage (phylum Cyanobacteria, class Vampirivibrionia, order and family Gastranaerophilales), while *Collinsella* was enriched within the family Coriobacteriaceae. In addition, several other genera—including *Odoribacter, Gordonibacter, RF39, Victivallis, Lactobacillus*, and *vadinBE97*—were more abundant in patients with high RBV according to LEfSe analysis (Figure 5C). Overall, these results suggest that while species‐level differences may be limited, genus‐level compositional shifts could reflect microbial associations with bladder function.

## 4 Discussion

Inflammation is a central mechanism in the pathogenesis of BPH, yet the initial trigger for this inflammatory process remains unclear. Recent evidence suggests that inflammation in distal organ systems, particularly the gut, may influence prostate pathology. In mouse models of chronic intestinal inflammation, prostate enlargement (PE) was observed and could be partially reversed by FMT from healthy donors or treatment with sodium butyrate, both of which reduce gut inflammation [38]. Mechanistically, certain gut bacteria appear to stimulate the release of proinflammatory cytokines and chemokines, promoting systemic and localized inflammation [39]. Growing evidence indicates that the gut and urogenital systems are functionally connected. Several studies have reported associations between gut microbial composition and prostate growth, BPH, and even prostate cancer. Individuals with PE exhibit higher relative abundances of Bacillota (Firmicutes) and Actinomycetota (Actinobacteria), but fewer Bacteroidota (Bacteroidetes), resulting in an elevated Bacillota‐to‐Bacteroidota ratio [20]. Animal studies further support these findings, showing that gut bacteria, such as Prevotellaceae and Bifidobacteriaceae, are involved in hormone and fatty acid metabolism, potentially linking microbial activity to prostate growth and function. Similarly, short‐chain fatty acid (SCFA)‐producing bacteria, including Rikenellaceae, *Alistipes*, and *Lachnospira*, have been associated with prostate cancer progression [19]. While most research has focused on prostate cancer, relatively little attention has been given to gut microbiome associations with BPH. In this study, we investigated gut microbial composition in relation to PV and RBV, providing exploratory insights into the emerging gut‐prostate axis.

In our cohort, we did not observe significant associations between PV and other clinical parameters, including age, BMI, alcohol consumption, smoking status, or RBV. However, PV correlated positively with PSA levels, consistent with established findings in the literature [32]. The absence of a clear age‐related association may be attributed to the relatively narrow age distribution of our participants, who largely fell within the demographic already at elevated risk for BPH [40]. Moreover, the generally low mean RBV values across the cohort suggest that surgical intervention was primarily indicated based on clinical symptoms rather than on uroflowmetry studies alone.

To assess whether overall gut microbial diversity was associated with prostate size and disease severity, we compared α‐ and β‐diversity metrics between patients grouped by PV and RBV. No significant differences were observed in microbial α‐ and β‐diversity between these subgroups. This suggests that specific compositional changes, rather than broad diversity shifts, are relevant to PE and bladder emptying dynamics. Our results parallel findings from prostate cancer research, where alterations in microbiome diversity indices have been inconsistent across studies [41, 42, 43, 44, 45, 46].

Although overall microbial diversity did not differ between groups, taxonomic‐level analyses revealed specific associations with clinical parameters. Notably, *Methanobrevibacter smithii* was less abundant in patients with larger PVs. As the predominant methanogenic archaeon in the human gut [47, 48], *M. smithii* is known to participate in anaerobic fermentation processes and methane production [49]. Alterations in *M. smithii* abundance may therefore reflect differences in intestinal fermentation capacity, including pathways related to short‐chain fatty acid (SCFA) metabolism; however, these functional outputs were not directly assessed in the present study [50, 51]. SCFAs, particularly butyrate, have been implicated in immune regulation and anti‐inflammatory processes in prior experimental and clinical studies, and systemic inflammation has been proposed as a contributing factor in prostate hyperplasia [21, 38, 52]. In this context, the observed association between *M. smithii* abundance and PV may be consistent with broader hypotheses linking gut microbial composition to inflammatory states, although any mechanistic interpretation remains speculative. Previous work has also reported associations between *M. smithii*, host energy balance, and obesity in animal models [36], and obesity itself has been associated with an increased risk of BPH [37]. However, in our cohort, *M. smithii* abundance did not significantly correlate with BMI, nor did BMI correlate with PV, likely reflecting the limited representation of obese individuals, as most participants had normal or moderately elevated body weight. Future studies incorporating direct measurements of microbial metabolites (e.g., methane and SCFAs) and inflammatory markers such as IL‐6 and TNF‐α will be necessary to determine whether these associations reflect functional or causal relationships [36, 50].

In addition to PV, we examined the gut microbiota in relation to RBV, a clinically relevant indicator of lower urinary tract function. Distinct microbial patterns were observed across RBV groups, with *Gastranaerophilales* showing higher abundance in patients with high RBV, whereas *Collinsella* was decreased in those with high RBV. Interestingly, the order Gastranaerophilales has previously been positively correlated with the risk of prostatitis and may play a causal role in increasing susceptibility to this condition [53, 54]. Given that a previous episode of prostatitis has been associated with an elevated risk of developing BPH and related complications [55, 56], the observed enrichment of Gastranaerophilales in patients with higher RBV may reflect shared disease‐associated or inflammatory contexts. A direct mechanistic link regarding common inflammatory pathways connecting gut microbial composition to prostatic or bladder dysfunction remains purely speculative. Conversely, *Collinsella* has been implicated in several metabolic and inflammatory conditions, including type 2 diabetes [57], hypercholesterolemia [58], nonalcoholic fatty liver disease [59], and rheumatoid arthritis [60], suggesting its potential role in metabolic dysregulation. Its abundance has also been shown to be diet‐sensitive, increasing particularly with low‐fiber intake [61]. Notably, *Collinsella* abundance has been reported to be decreased in prostate cancer and has been proposed as a potential biomarker for its detection or prognosis [62]. In our study, the lower *Collinsella* abundance observed in patients with higher RBV may reflect context‐specific associations that warrant further investigation. Overall, these findings suggest exploratory associations between gut microbial composition and RBV that warrant further investigation. Future studies incorporating longitudinal designs and functional measurements will be required to clarify whether these taxa play context‐specific roles in urogenital physiology or disease.

This study has several limitations. First, the small, cross‐sectional sample size reduces statistical power and precludes causal inference. Second, several important confounders known to influence gut microbiota composition were not systematically assessed, including dietary intake, probiotic or supplement use, bowel habits, metabolic syndrome‐related parameters, and detailed medication history, which may introduce residual confounding. Future studies should adopt larger, longitudinal designs and incorporate functional readouts such as metabolomics or direct measurements of SCFAs and inflammatory markers to better contextualize observed taxonomic associations. Long‐term research directions could also explore microbiome‐modulating interventions, such as FMT or prebiotics, to examine their potential effects on LUTS and prostate size. Additionally, the identification of reproducible microbial biomarkers may provide a foundation for future hypothesis‐driven studies in BPH risk stratification. An important consideration for future research is the timing of measurements: longitudinal studies are required to determine whether microbiome signatures precede BPH development or are associated with clinically relevant LUTS.

## 5 Conclusion

In conclusion, our study identifies exploratory associations between specific gut microbial taxa and prostate health, as well as lower urinary tract function, supporting the concept of a gut‐prostate axis. *Methanobrevibacter smithii* was linked to PV, while *Collinsella* and *Gastranaerophilales* were associated with RBV, suggesting that microbial composition may reflect both anatomical and functional aspects of BPH. While causality cannot be established, these findings offer a foundation for future research aimed at elucidating microbial mechanisms and assessing whether microbiome features could serve as biomarkers or inform long‐term experimental investigations into prostatic disease.

## Author Contributions

Conceptualization: Uwe Bieri and Cédric Poyet. Methodology: Jonathan Surber, Marie Lork, Yasser Morsy, Uwe Bieri, and Cédric Poyet. Formal analysis: Jonathan Surber, Marie Lork, and Yasser Morsy. Investigation: Jonathan Surber and Marie Lork. Resources: Michael Scharl and Daniel Eberli. Data curation: Jonathan Surber, Marie Lork, and Uwe Bieri. Writing – original draft: Jonathan Surber, Marie Lork, and Uwe Bieri. Writing – review and editing: Jonathan Surber, Marie Lork, Yasser Morsy, Michael Scharl, Daniel Stephan Engeler, Janine Langenauer, Lukas John Hefermehl, Anna Ebner, Basil Kaufmann, Manuela Hunziker, Daniel Eberli, Uwe Bieri, and Cédric Poyet. Visualization: Jonathan Surber, Marie Lork, and Yasser Morsy. Supervision: Uwe Bieri and Cédric Poyet. Funding acquisition: Uwe Bieri and Cédric Poyet.

## Ethics Statement

All patients enrolled in the SILENT‐EMPIRE study signed a general consent for the storage of their biological material in a biobank and for future research use (BASEC. 2021‐01783). For the present study, a separate Ethics Committee‐approval was obtained (BASEC. 2024‐02403, date: June 20, 2025). The study was conducted in accordance with the Declaration of Helsinki and Good Clinical Practice guidelines.

## Conflicts of Interest

The authors declare no conflicts of interest.

## Supporting information


**Figure S1:** Metagenomic landscape and age‐associated differences in gut microbial abundances. (A‐B) Descriptive visualization of microbial taxonomic composition at the phylum (A) and genus (B) levels, illustrating distinct microbial profiles across individual patients. Only the 10 most abundant phyla and 20 most abundant genera across the cohort are annotated in the legend. (C‐D) Relative abundance of Butyricicoccus (C) and Ruminococcus (D) in participants aged ≥ 65 years (n = 15) versus < 65 years (n = 13). Box plots show minimum to maximum values with median. Statistical significance was assessed using the Mann‐Whitney test.

**Table S1:** Summary of α‐ and β‐diversity metrics in relation to PV and RBV.

**Table S2:** Absolute abundance of bacterial taxa.

## Data Availability

Data is provided within the manuscript or supplementary information files.
