# Supplementary figures and images for: Exploring the Gut‐Prostate Axis: Microbial Signatures Linked to Prostate Volume and Bladder Function
Source: Prostate. 2026 Mar 18;86(8):900–10. doi: 10.1002/pros.70160 (PMC13116016; doi:10.1002/pros.70160)

FigureS1

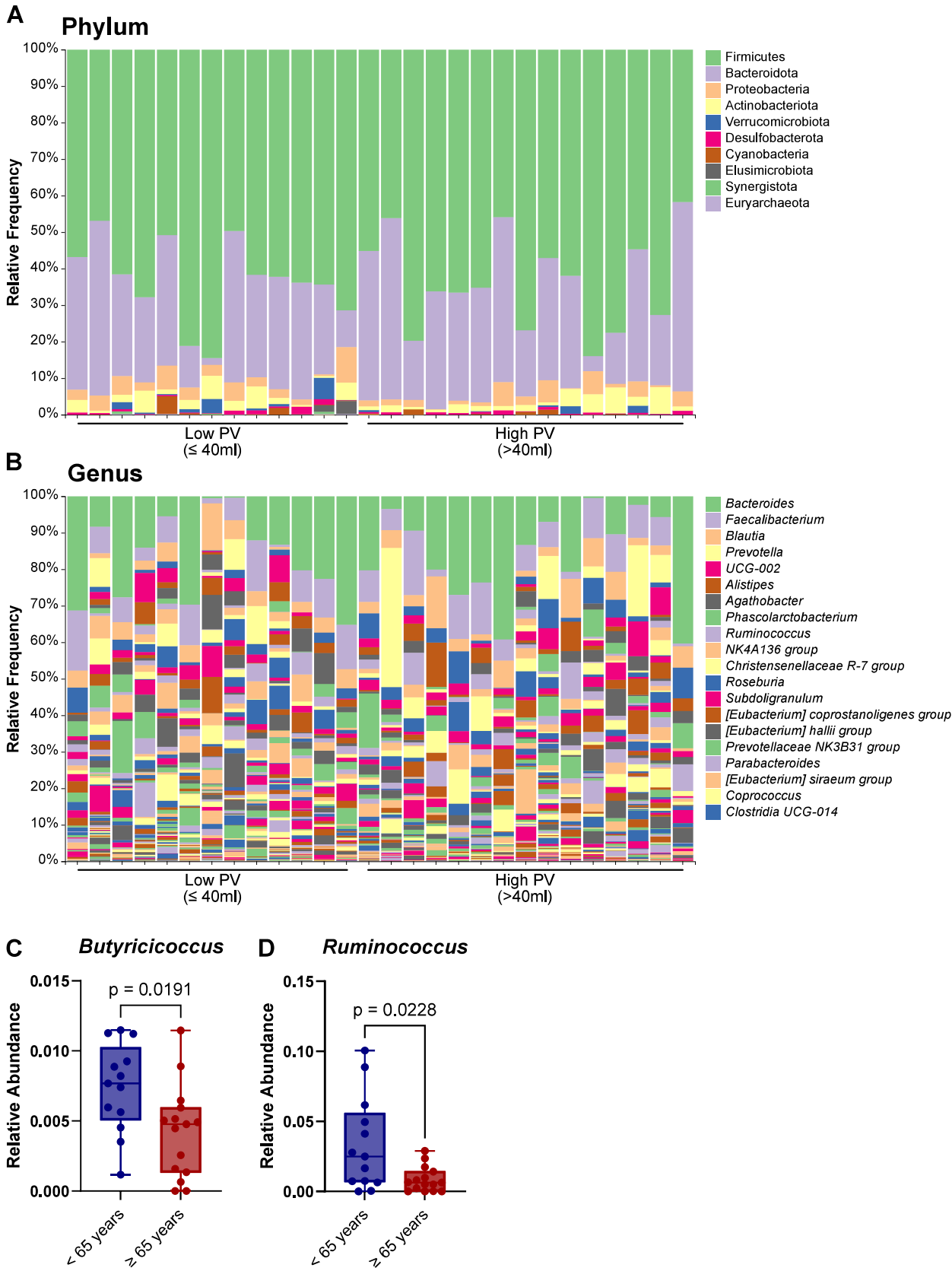

Supplement: Supplementary file 1 — Figure S1: Metagenomic landscape and age‐associated differences in gut microbial abundances. (A‐B) Descriptive visualization of microbial taxonomic composition at the phylum (A) and genus (B) levels, illustrating distinct microbial profiles across individual patients. Only the 10 most abundant phyla and 20 most abundant genera across the cohort are annotated in the legend. (C‐D) Relative abundance of Butyricicoccus (C) and Ruminococcus (D) in participants aged ≥ 65 years (n = 15) versus < 65 years (n = 13). Box plots show minimum to maximum values with median. Statistical significance was assessed using the Mann‐Whitney test. [file PROS-86-900-s003.pdf]
